# Melatonin: Current status and future perspectives in horticultural plants

**DOI:** 10.3389/fpls.2023.1140803

**Published:** 2023-03-23

**Authors:** Jing Zhao, Junjie Hu

**Affiliations:** School of Agricultural, Jilin Agricultural Science and Technology University, Jilin, China

**Keywords:** root architecture, ion homeostasis, melatonin, redox balance, horticultural crops

## Abstract

Global warming in this century increases incidences of various abiotic stresses, restricting plant growth and productivity and posing a severe threat to global food production and security. Different phytohormones are produced by plants to mitigate the adverse effects of these stresses. One such phytohormone is melatonin (MEL), which, being a potential bio-stimulator, helps to govern a wide array of functions in horticultural crops. Recent advancements have determined the role of MEL in plants’ responses to abiotic stresses. MEL enhances physiological functions such as seed germination, growth and development, seedling growth, root system architecture, and photosynthetic efficiency. The potential function of MEL in stressful environments is to regulate the enzymatic and non-enzymatic antioxidant activity, thus playing a role in the substantial scavenging of reactive oxygen species (ROS). Additionally, MEL, as a plant growth regulator and bio-stimulator, aids in promoting plant tolerance to abiotic stress, mainly through improvements in nutrient uptake, osmolyte production, and cellular membrane stability. This review, therefore, focuses on the possible functions of MEL in the induction of different abiotic stresses in horticultural crops. Therefore, this review would help readers learn more about MEL in altered environments and provide new suggestions on how this knowledge could be used to develop stress tolerance.

## Introduction

**Figure 1:**
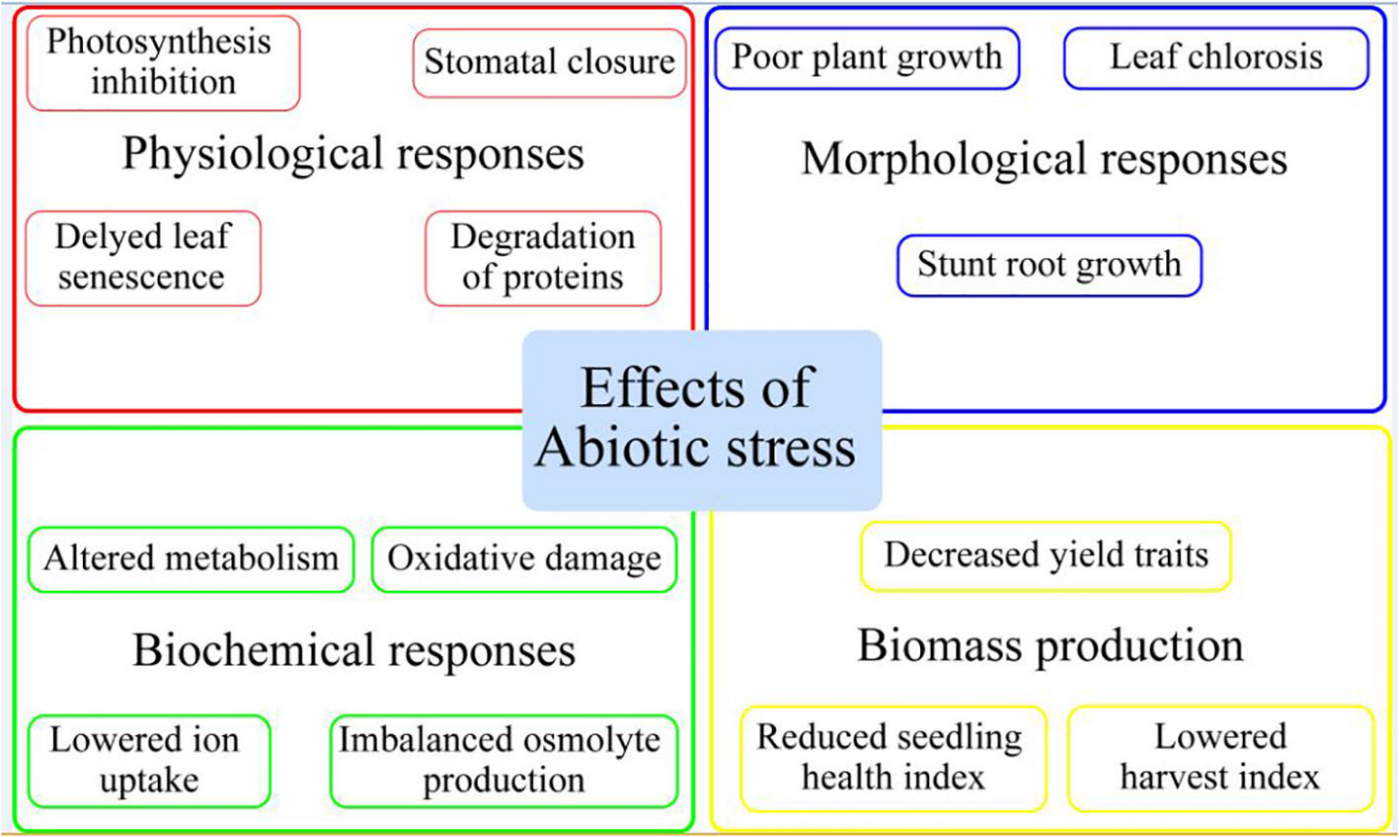
Effect of abiotic stress on horticultural plants.

Plants, being sessile organisms, face a variety of environmental stresses (low and high temperature, metal stress, salinity, and drought stress) (Hassan et al., 2022), which have detrimental impacts on their performance in terms of growth and development (Rasheed et al., 2021; Altaf et al., 2023). It has been projected that about 90% of arable land is susceptible to one of the above-mentioned stresses (Dos Reis et al., 2012). Due to the devastating impact on the growth and productivity of agricultural crops, global attention has been diverted to these abiotic stresses. Various developmental functions and processes of plants, including morphological, physiological, and biochemical, are disrupted by these abiotic stresses (Marino, 2021; Arnao et al., 2023). Further, environmental stresses cause significant yield losses through excessive production of reactive oxygen species (ROS), nutrient deficiencies, decrease in photosynthetic efficiency, reduction in root growth, and osmolyte over-accumulation (**Figure 1**) (Ayyaz et al., 2022; Imran et al., 2022). With the ongoing changes in climate, these abiotic stresses are getting intensified, thus calling for the need for appropriate controlling measures (Gao et al., 2007; Andreotti, 2020; Shahid et al., 2021). In horticultural crops such as tomato, potato, pepper, and cucumber, around 70% of total yield losses are due to effects caused by environmental stresses at different growth phases (Martinez et al., 2018; Zörb et al., 2018). To promote sustainable agriculture, different management strategies have been introduced for achieving the targets (Ahammed and Li, 2023). Such techniques include plant growth regulators, different osmolyte syntheses, and accumulation to protect against stress-induced damages for maintaining cellular homoeostasis and optimum plant growth (Nawaz et al., 2017; Koza et al., 2022; Peng et al., 2023).

**Figure 2:**
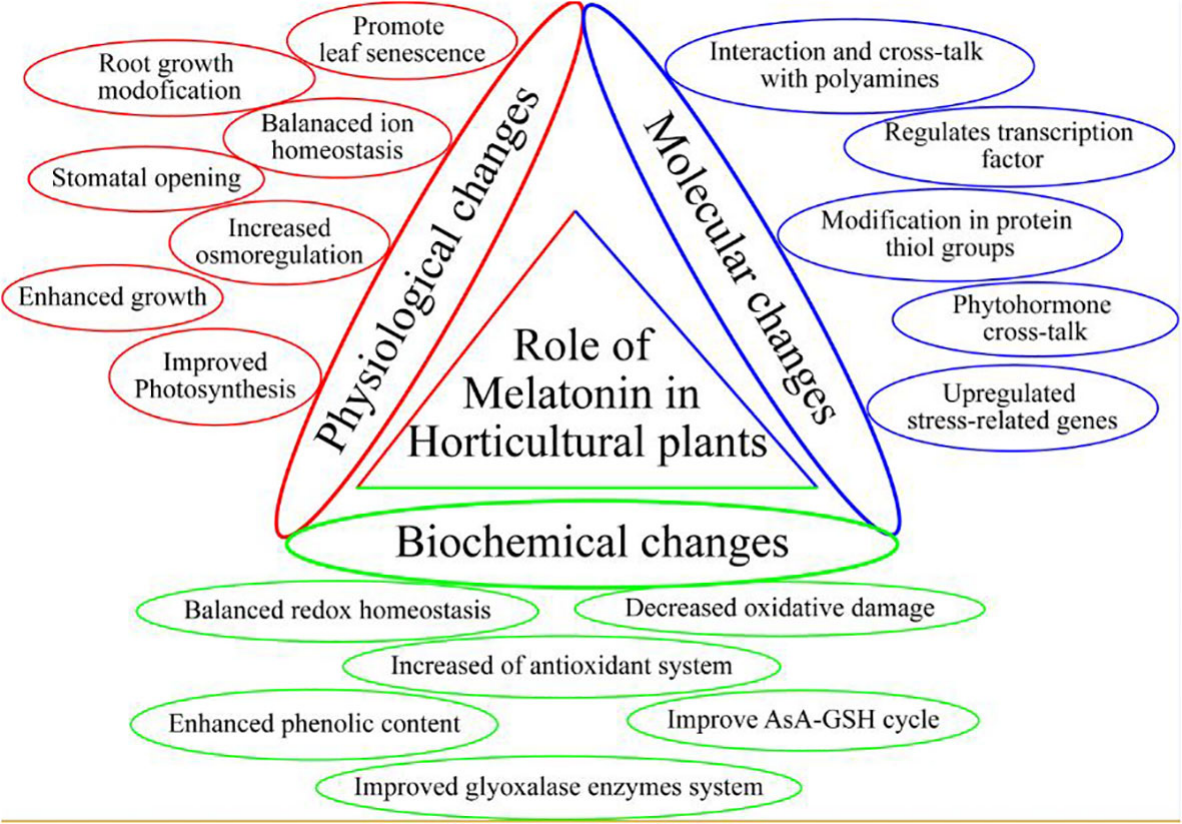
Role of melatonin in horticultural plants.

One of the essential plant growth regulators in stressed environments is melatonin (MEL), which is a small molecule acting as a powerful antioxidant, thus enhancing the stress resistance of plants against many environmental stressors (Hoque et al., 2021) (**Table 1**). This pleotropic molecule is found in various plant parts of several plant species, such as broccoli, coriander, mango, cabbage, tobacco, cucumber, and orange (Badria, 2002; Posmyk et al., 2009; Johns et al., 2013; Aguilera et al., 2015). MEL is also involved in the regulation of seed germination, seedling growth, photosynthetic efficiency, root system architecture, leaf senescence, fruit ripening, stomatal opening, and redox homeostasis (**Figure 2**) (Jan et al., 2022). Further, MEL has been well defined as an anti-stress promoter and growth bio-stimulator for horticultural plants, particularly in adverse environmental situations, such as cold, heat, heavy metals, salinity, drought, acidic rain, and UV stress (Sharif et al., 2018; Aghdam et al., 2021; Wu et al., 2021; Zhao et al., 2022). Plants are protected against stressful environments by the regulation of gene expressions mediated by MEL, such as the plants’ “antioxidant defense system” activation (Jahan et al., 2020) which places MEL among vital bio-stimulants to improve crop productivity in stress conditions. In stressful environments, MEL helps to trigger the antioxidant defense system, which favors the scavenging of ROS and thus acts as a stress protector (Moustafa-Farag et al., 2020). Due to this function, MEL is a promising molecule that can be applied exogenously to alleviate stress. The current review aims to explore the biochemical and physiological functioning of MEL in abiotic stress environments, along with its possible mechanism of action. Further, the future aspect of MEL-regulated stress tolerance of horticultural crops is also discussed for a detailed overview of the research.

**Table 1:** Exogenous melatonin enhanced abiotic stress tolerance in horticultural plants.

Stress type	MEL levels	Scientific name	Reference
Cold	5 µM	*Capsicum annum* L.	Korkmaz et al. (2021)
	100 µM	*Solanum lycopersicum* L.	Ding et al. (2017)
	200 µM	*Cucumis sativus* L.	Zhao et al. (2017)
	150 µM	*Citrullus lanatus* L.	Li et al. (2017b)
	5 µM	*Solanum melongena* L.	Yakuboğlu et al. (2022)
	200 µM	*Cucumis melo* L.	Zhang et al. (2017c)
	100 µM	*Prunus persica*	Gao et al. (2018)
Heavy metal	100 µM	*Capsicum annum* L.	Kaya et al. (2022)
	100 µM	*Fragaria × ananassa* Duch.	Wu et al. (2021)
	0.1 µM	*Citrullus lanatus* L.	Nawaz et al. (2018)
	100 µM	*Nicotiana tabacum* L.	Wang et al. (2019)
	100 µM	*Spinacia oleracea* L.	Asif et al. (2020)
Salinity	1 µM	*Solanum lycopersicum* L.	Ali et al. (2021)
	150 µM	*Citrullus lanatus* L.	Li et al. (2017a)
	50–150 µM	*Cucumis sativus* L.	Wang et al. (2016)
	100 and 200 µM	*Fragaria × ananassa* Duch.	Zahedi et al. (2020)
	0.1 µM	*Malus hupehensis*	Li et al. (2012)
	50 µM	*Vitis vinifera* L.	Xu et al. (2019)
Drought	100 µM	*Cucumis sativus* L.	Zhang et al. (2013)
	100 µM	*Solanum lycopersicum* L.	Liu et al. (2015a)
	100 µM	*Actinidia chinensis*	Liang et al. (2019)
	100 µM	*Carya cathayensis*	Sharma et al. (2020)
	0.2 µM	*Vitis vinifera* L.	Meng et al. (2014)
	100 µM	Camellia sinensis L.	Li et al. (2019)
Heat	100 µM	*Solanum lycopersicum* L.	Ahammed et al. (2018)
	100 µM	*Cucumis sativus* L.	Zhang et al. (2013)
	100 µM	Apium Graveolens L.	Li et al. (2022b)
	100 µM	*Fragaria × ananassa* Duch.	Manafi et al. (2022)

## Function of melatonin in horticultural crops

**Figure 3:**
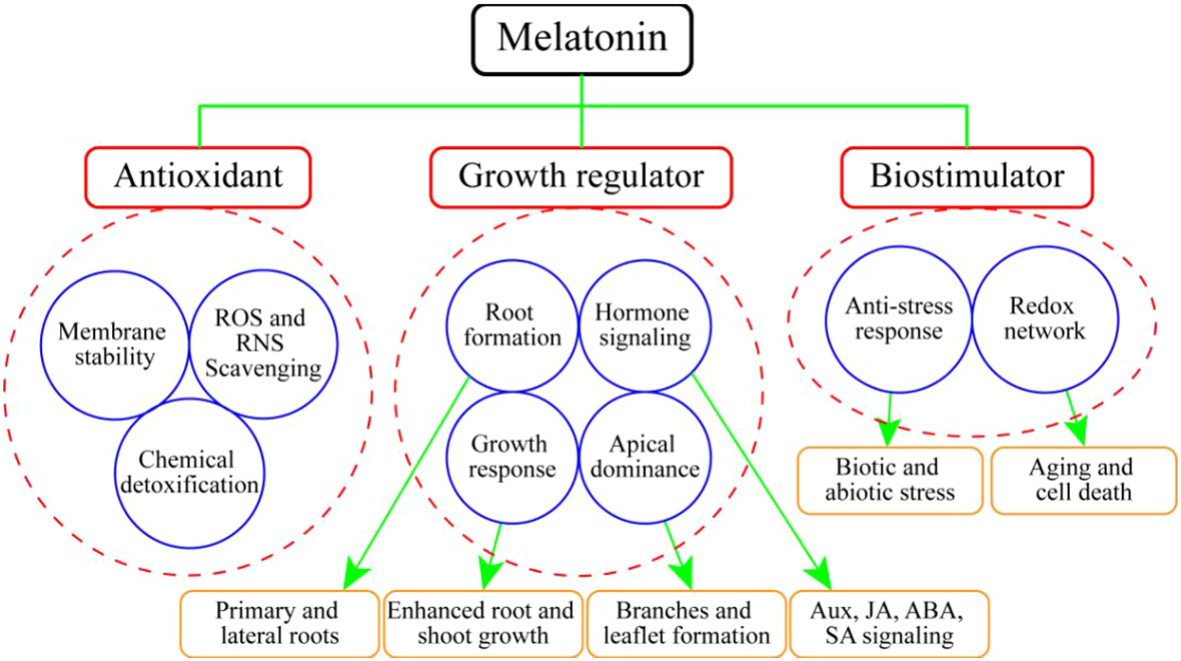
Action of melatonin inhorticultural plants as an antioxidant, growth regulator and bio-stimulator.

MEL promotes plant growth and development *via* different functions, most of which are related to different abiotic stressors such as drought, temperature fluctuation, heavy metals, and salinity (Arnao and Hernández-Ruiz, 2006; Altaf et al., 2022a). MEL functions as a growth regulator, bio-stimulator, and potential antioxidant compound (Arnao and Hernández-Ruiz, 2014) (**Figure 3**). A primary function attributed to MEL in plants is to act as an antioxidant, providing protection against environmental agents (Nawaz et al., 2016; Tiwari et al., 2022). However, one important function of MEL may be the scavenging of free radicals, thereby protecting plants against oxidative damage (Paredes et al., 2009). MEL is significantly involved in the process of leaf senescence (Arnao and Hernández-Ruiz, 2009). A range of different functions of MEL have been investigated in horticultural plants, some more thoroughly than others, but in all cases the data are scarce (**Table 2**).

**Table 2:** Function of melatonin in horticultural plants.

Functions	Reference
Improved seed germination	Zhang et al. (2013)
Regulation of circadian rhythms	Kolář and Macháčková (2005)
Modification of root system architecture	Nawaz et al. (2018)
Vegetative development	Tan et al. (2013)
Regulation of photosynthetic machinery	Jahan et al. (2021)
Role as a potential growth regulator	Murch and Saxena (2002)
Enhanced seedling health index	Liu et al. (2015a)
Protection against environmental stresses	Ali et al. (2021)
Balanced of mineral nutrient homeostasis	Sarafi et al. (2017)
Reproductive development	Arnao and Hernández-Ruiz (2006)
Fruit ripening	Arnao and Hernández-Ruiz (2020)
Maintenance of ROS homeostasis	Siddiqui et al. (2020b)
Cell protection	Ahammed et al. (2020)
Regulation of antioxidant enzymes pool	Zamani et al. (2020)
Retardation of leaf senescence	Shi et al. (2015)
Modulation of flowering development	Paredes et al. (2009)
Inhibition of root elongation	Park and Back (2012)

## Melatonin as abiotic stress regulator in horticultural crops

### Salinity

Salinity has been declared a significant hazard in modern horticulture, as it impairs and inhibits the growth and development of plants, mainly through disruption of the soil’s osmotic and ionic balances (Abdelaal et al., 2020; Zulfiqar et al., 2022). With increments in soil salt levels, an osmotic stress condition develops, which leads to declining water levels in the soil, thus less water is available for plant uptake, causing a conditional physiological drought in plants (Chang et al., 2014). Recently, MEL has emerged as an effective plant growth regulator, playing a significant role in the development of abiotic stress resistance in horticultural crops. Resultantly, several studies highlighted the stress-mitigating functions of MEL in horticultural crops under salt stress (Bose and Howlader, 2020). Plants’ antioxidant system has been reported to improve, and photosynthetic capacity is seen to get protected with exogenously applied MEL under NaCl stress in peanut (ElSayed et al., 2020), orange (Hu et al., 2022), watermelon (Li et al., 2017a), pistachio (Kamiab, 2020), and tomato (Liu et al., 2019). According to Li et al. (2012), the photosynthetic capacity of plants is maintained by the exogenous application of MEL (0.1 µM) under salinity stress, which leads to significant alleviation of growth inhibition. Further, the oxidative damage caused by the scavenging of ROS was also decreased by MEL, and antioxidant enzymes’ activity was improved, including catalase, peroxidase, and ascorbate. Salinity exerts its negative impact irrespective of the growth stage of the plants, and its effects range from seed germination to plant senescence and occur throughout the life cycle. Seed germination and plant growth are severely affected by saline stress (Nawaz et al., 2016; Zhan et al., 2019). In tomato, under salinity stress, the MEL applied exogenously reduced the uptake of sodium (Na^+^), hydrogen peroxide (H_2_O_2_) content, and malonaldehyde (MDA) content, while enhancing enzyme activity, relative water content (RWC), membrane stability index, gas exchange parameters, and growth attributes (Ali et al., 2021). Furthermore, MEL pre-treatment of cucumber seeds showed an increase in seed germination rate and seedling growth, along with a 5-fold increase in antioxidant enzyme activity under salinity stress (Zhang et al., 2014).

MEL supplementation improved growth traits and reduced the levels of MDA, ROS, and EL (electrolyte leakage), mainly through upregulating the enzymatic and non-enzymatic antioxidant enzyme activity. Moreover, in strawberries, MEL improved the phenolic and photosynthetic content (Zahedi et al., 2020). Importantly, Hu et al. (2021) revealed efficient reductions in the levels of MDA and ROS, increases in antioxidant activities, endogenous levels of MEL, proline, and pigment content, stomatal conductance, and the upregulation of genes related to redox, salt tolerance, and MEL biosynthesis. In addition, MEL was seen to escalate the ion homeostasis under high-NaCl stress in *Malus hupehensis* (Li et al., 2012). MEL further reduced ion toxicity by suppressing the accumulation of Na^+^ and Cl^−^ ions (Liu et al., 2019). The tomato seedling growth showed significant improvements with the supplementation of MEL under NaCl toxicity. Additionally, MEL effectively reduced the activity of glycolate oxidase, chlorophyll degradation, and ROS levels and caused increases in antioxidant enzyme activity, proline content, and glycine betaine levels (Siddiqui et al., 2020a). According to Zhang et al. (2017a), the uniformity of seeds and germination rate of cucumber seeds increased with the regulation of energy production with the application of MEL under salinity stress. Additionally, MEL protects the photosynthetic apparatus from oxidative damage induced by NaCl stress (Zhang et al., 2020). MEL increases the antioxidant enzyme, thus leading to a decline in the accumulation of ROS in the leaves of salt-sensitive cucumber plants. In cucumber, MT was also reported to suppress the alleviation in maximum quantum efficiency of photosystem II (PSII) and net photosynthetic rate and to protect the total chlorophyll content under salinity stress (Wang et al., 2016).

### Drought

Global climate change has intensified drought stress episodes, which are emerging as a serious threat to crop growth and productivity worldwide. Horticultural crops are very vulnerable to drought stress (Gao et al., 2022; Muhammad et al., 2022; Toscano et al., 2023). Drought stress is reported to cause abnormalities in the physiological and morphological states of plants (Hanaka et al., 2021). Reduced root system architecture, cellular membrane integrity, damaged photosynthetic apparatus, and imbalanced mineral and nutrient accumulation are highlighted as some of the most important abnormalities that lead to the complete devastation of a plant facing drought stress (Tabassum et al., 2021). Melatonin protects horticultural crops by preventing damage to the root architecture system, photosynthetic machinery, inducing the antioxidative defense system, regulating oxidative stress, and some other defense mechanisms (Tiwari et al., 2020). MEL pretreatment of tomato seedlings grown in field conditions under drought stress showed effective results, and the detrimental effects of drought stress were significantly reduced (Liu et al., 2015a). MEL-supplementation effectively improved seedling growth, photosynthetic efficiency, activity of antioxidant enzymes, and decreased oxidative damage (Liu et al., 2015a). Under drought stress, exogenous MEL application improved the growth, photosynthetic apparatus, and antioxidant enzyme systems of Chinese hickory plants (Sharma et al., 2020). In addition, Karaca and Cekic (2019) observed that MEL supplementation efficiently improved chlorophyll content, antioxidant enzyme systems, and reduced MDA content in *Solanum lycopersicum* L. under drought stress.

The photosynthetic machinery of tomato seedlings under drought stress showed significant improvements with the application of MEL (Ding et al., 2018; Ibrahim et al., 2020). Similar kinds of studies that indicate the impact of melatonin treatments on minimizing drought-induced photosynthetic damage have been performed on fenugreek (Zamani et al., 2020), kiwifruit (Xia et al., 2020), and grapes (Meng et al., 2014). Exogenous melatonin treatment showed several positive phenomena, such as preventing chloroplast photosynthetic damage (Wang et al., 2012). Further, MEL supplementation led to improved turgor pressure and water content of leaves, along with enacting the spongy tissue (Meng et al., 2014). The antioxidant defense system of plants gets triggered, and the scavenging of ROS is efficiently enhanced by the application of MEL. In horticultural plants, the mechanisms related to these phenomena are well examined, such as the scavenging of H_2_O_2_ by the regulation of the ascorbate-glutathione cycle (Li et al., 2019; Ibrahim et al., 2020). In cucumber, the seed germination rate was improved by the exogenous application of MEL (Zhang et al., 2013). In *Moringa oleifera*, the foliar application of MEL under drought stress showed a beneficial impact on the dry and fresh weight of shoots and leaves, number of leaves/plants, plant height, and foliage yield (Sadak et al., 2020). (Sadak et al., 2020). In *Coffea arabica* seedlings, the leaf area was reduced by drought stress, which was alleviated by the supplementation of MEL (Campos et al., 2019). Plant growth is maintained by the application of MT under drought stress conditions, mainly by the maintenance of homeostatic balance and vegetative tissues’ growth (Sharma and Zheng, 2019). The protective roles of MEL in plants under salinity and drought stress are summarized in **Table 3**.

**Table 3:** Melatonin enhanced salinity and drought stress tolerance in horticultural crops.

Species name	Stress treatment	MEL level	Observation	References
Salinity stress				
Strawberry	0, 40, and 80 mM	100 and 200 μM	Enhanced strawberry fruit production, phenolics content, photosynthetic pigment, decreased oxidative stress biomarkers, and boost antioxidant enzymes system.	Zahedi et al. (2020)
Orange	150 mM	0, 50, 100, 150 µM	Better plant growth, enhanced photosynthetic efficiency, and pigment content	Hu et al. (2022)
Cucumber	150 mM	50, 100, 200, 300 μM	Improved photosynthesis, growth, carotenoids, and antioxidant enzymes; reduced MDA, EL, and H_2_O_2_ level	Zhang et al. (2020)
Pistachio	25, 50, 100, 150 mM	0, 25, 50, 75, 100, 125, 150 μM	Enhanced shoot and root growth, chlorophyll content, polyamine level, proline content, nutrient uptake, and antioxidant enzymes activity	Kamiab (2020)
Tomato	160 mM	1.0 µmol	Increased RWC, growth traits, gas exchange characteristics, pigments content, and antioxidant enzymes system; reduced MDA and H2O2 level; decreased Na^+^ accumulation	Ali et al. (2021)
Peanut	150 mM	50, 100, 150 μM	Reduced ROS, and MDA level, increased enzymatic and non-enzymatic antioxidant system and proline content	ElSayed et al. (2020)
Watermelon	300 mM	50, 150, and 500 μM	Reduced oxidative damage, balanced redox homeostasis, protect photosynthesis, and upregulate antioxidant enzyme system	Li et al. (2017a)
Drought				
Kiwifruit	9 days	50, 100, and 200 μM	Modified root system architecture, decreased MDA level, enhanced osmoregulation content, increased photosynthesis	Liang et al. (2019)
Fenugreek	7 days	50, 100, 300, and 500 μM	Improved growth parameters, Enhanced antioxidant enzymes and proline content; reduced H_2_O_2_ and MDA level	Zamani et al. (2020)
Cucumber	–	100 μM	Reduced ROS production, decreased chlorophyll degradation, increased net photosynthetic rate	Zhang et al. (2013)
Tomato	–	100 μM	Promoted growth, chlorophyll content, proline level, soluble sugar content, and antioxidant enzymes system	Ibrahim et al. (2020)
Tobacoo	14 days	200 μM	Promote root system architecture, enhanced nutrient uptake and antioxidant enzymes system, reduced H2O2 and MDA level	Liu et al. (2021)

### Cold

Plant growth is significantly influenced by temperature. Low temperature stress lies amid the most detrimental environmental conditions for plants, resulting in yield and productivity losses (Malhotra, 2017). Cold stress can negatively affect seedling growth, root morphology, photosynthetic efficiency, seed germination, and pigment content (Marino, 2021). Low-temperature stress also has a negative impact on the metabolic capacity of plants. Plants’ metabolic capacity is also affected by low-temperature stress. The levels and activity of enzymes involved in important metabolic pathways are usually altered in cold-stressed plants, and as a result, the plant metabolome is completely changed (Khan et al., 2015; Liang et al., 2020). The development of commercial crop cultivars that are cold-resistant has been recently focused on by plant scientists. The use of MEL significantly reduced the adverse effects of cold stress on a diverse range of plant genera. Wang et al. (2020) reported that MEL application remarkably improved photosynthesis, metabolites, and tomato seedling growth under cold stress. MEL application efficiently improved photosynthetic performance in pepper under chilling stress (Korkmaz et al., 2021). In tomato seedlings, the application of MEL under cold stress caused significant increases in chlorophyll fluorescence parameters, pigment content, gas exchange elements, and growth characteristics (Zhou et al., 2020). Furthermore, melatonin significantly imparted cold tolerance in *Citrullus lanatus* stemically by regulating antioxidant capacity and the expression of defense genes (Li et al., 2017b). In a recent study, Li et al. (2022a) reported that MT supplementation efficiently enhanced root growth, antioxidant enzymes, the photosynthetic system, and reduced oxidative damage in pepper under cold stress.

MEL supplementation increased seedling growth and cold stress tolerance by balancing redox homeostasis, stomatal opening, leaf photosynthetic activity, mineral nutrient accumulation, osmolytes production, and primary and secondary metabolites, as well as improvements in antioxidant activities and ROS scavenging (Qari et al., 2022). MEL alleviates cold-induced adverse effects on tea plants. MEL pretreatment in tea plants significantly improved growth traits, photosynthesis, antioxidant profile, and balanced redox homeostasis under chilling stress (Li et al., 2018). Root pretreatment with MEL reduced aerial cold-induced suppression of photosystem II and oxidative damage in *C. lanatus* (Chang et al., 2020). Cao et al. (2016) revealed that in peach fruit, chilling injury was very effectively reduced by the application of MEL at a dose of 100 μM. In tomato plants under cold stress, the pretreatment of MEL showed higher levels of non-enzymatic antioxidants, greater activities of antioxidant enzymes, and reduced MDA content and EL (Ding et al., 2017). Furthermore, pretreatment of MEL declined the harmful impact of cold stress and accelerated the plants’ recovery, primarily by improving photosynthesis and antioxidant enzyme capacity in the leaves of melon (Zhang et al., 2017b). Several reports revealed that MEL positively modulates the growth of cucumber (Marta et al., 2016), tomato (Yang et al., 2018), and watermelon (Li et al., 2017a). MEL application considerably reduced the adverse effect of cold stress on eggplant seedlings. MEL supplementation enhanced leaf area, biomass production, photosynthetic mechanism, activity of antioxidant enzymes, proline content, and reduced MDA and H_2_O_2_ levels of eggplant seedlings under chilling conditions (Yakuboğlu et al., 2022).

### Heat

The production of horticultural crops is severely and significantly hindered by heat stress driven by climate change. High temperature stress is a major environmental stress that limits plant growth, metabolism, and productivity worldwide. Temperature affects many of the biochemical reactions that are important for the steady growth and development of plants (Hasanuzzaman et al., 2013). High temperatures are becoming an important concern for sustainable crop production (Wahid et al., 2012). A few researchers have investigated the possible role and defensive mechanism of MEL under heat stress in plants. Recently, Ahammed et al. (2018) revealed that MEL has been declared a universal regulator of abiotic stresses, which can possibly increase the heat resistance of plants. MEL-pretreated tomato plants under heat stress showed improved root growth, chlorophyll content, activities of enzymatic and non-enzymatic antioxidants, and decreased oxidative damage. Further, Jahan et al. (2019) described that MEL efficiently increased polyamine content and considerably declined levels of MDA and EL in tomatoes. MEL supplementation effectively reduced the accumulation of ROS and increased the antioxidant profile in tomato seedlings in a high-temperature environment (Martinez et al., 2018). Ahammed et al. (2019) revealed that MEL supplementation significantly reduced MDA and EL levels and increased the antioxidant enzyme system in tomatoes under heat stress. **Table 4** shows how MEL protects plants from heat and cold stress. Research to date has demonstrated the vital functions of MEL for plant survival and higher productivity of horticultural crops in high-temperature stress conditions. Yet, extensive investigations are needed to confirm the possible mechanisms of heat stress amelioration by MEL in plants.

**Table 4:** Action of melatonin in cold and heat stress tolerance.

Species name	Stress treatment	MEL level	Observation	References
Cold stress				
Pepper	10/4°C D/N	5 μM	Improved chlorophyll content, gas exchange characteristics	Altaf et al. (2022b)
Peach	4°C	100 µM	Reduced H_2_O_2_ level and enhanced antioxidant enzymes system	Cao et al. (2018)
Cucumber	15/8°C D/N	200 μM	Decreased ROS production and upregulated AsA-GSH cycle,	Zhao et al. (2016)
Tomato	15/6°C D/N	100 µM	Enhanced photosynthetic machinery, reduced MDA accumulation	Yang et al. (2018)
Melon	12/6°C D/N	200 μM	Improved chlorophyll content and gas exchange parameters, lowered MDA level and increased antioxidant enzymes system	Zhang et al. (2017c)
Banana	4°C	100 µM	Enhanced ETR, improved antioxidant enzymes system; reduced MDA and H_2_O_2_, O_2_ accumulation	Liu et al. (2022)
Heat stress				
Tomato	42°C	100 µM	Lowered ROS accumulation, reduced MDA production, enhanced enzymatic and non-enzymatic antioxidant system	Jahan et al. (2019)
Kiwifruit	45°C	200 μM	Enhanced AsA-GSH cycle, proline content antioxidant enzymes system, and reduced oxidative damage	Liang et al. (2018)
Radish	35/30°C D/N	29.0 mg	Enhanced biomass yield, antioxidant enzymes, carotenoids content	Jia et al. (2020)
Celery	38°C	100 µmol	Reduced EL and MDA level; enhanced chlorophyll content	Li et al. (2022b)

### Heavy metals

Globally, the pollution caused by heavy metals is getting worse with time, causing a wide range of toxic impacts on horticultural crop production (Shakoor et al., 2017; Behera et al., 2022). Plants are probably universally tolerant of heavy metal stress. Regardless of other stresses, the production of horticultural crops is significantly and negatively affected by heavy metal stressors, which is becoming a major concern (Noor et al., 2022). Hitherto work shows that heavy metal (lead, boron, cadmium, nickel, arsenic, and vanadium) stress remarkably reduces horticultural crop production (Dodangeh et al., 2018; Bhat et al., 2019).

One of the recently emerging potential stress-alleviating hormones is MEL, which may aid in coping mechanisms against metal-induced toxicity in plants. MEL application remarkably increased growth characteristics, root morphology, pigment content, and net photosynthetic rate. Additionally, under vanadium stress, MEL application in watermelon caused significant reductions in oxidative damage, increases in antioxidant enzymes, and levels of relevant gene expression (Nawaz et al., 2018). Under nickel toxicity, MEL application in tomato reduced the MDA and EL levels and increased the secondary metabolite content, proline level, leaf photosynthesis, and antioxidant defense mechanism (Jahan et al., 2020). MEL application considerably increased the root architecture of cucumber seedlings under copper toxicity (Cao et al., 2019), the growth status of red cabbage plants under copper toxicity (Posmyk et al., 2008), the photosynthetic efficiency of radish seedlings under aluminum toxicity (Tang et al., 2016), the antioxidant enzyme system in roses under cadmium toxicity (Nabaei and Amooaghaie, 2019), the mineral nutrient content of strawberry seedlings under cadmium toxicity (Wu et al., 2021), and lowered the MDA and EL levels in tomato seedlings under cadmium toxicity (Hasan et al., 2015). Moreover, previous findings suggested that MEL significantly reduced iron accumulation from root to shoot in cucumber, watermelon, and tomato (Ahammed et al., 2020).

Hasan et al. (2018) revealed that under low sulfur conditions, MEL efficiently enhanced different growth traits, chlorophyll fluorescence parameters, gas exchange elements, and pigment molecules, as well as declining the MDA and H_2_O_2_ levels in tomato seedlings. Siddiqui et al. (2019) observed that MEL application significantly improved the photosynthesis and growth of tomato seedlings under lanthanum toxicity. Further, MEL supplementation significantly improved aerial biomass production, carotenoid content, chlorophyll content, and carbohydrate levels in spinach under boron toxicity. Selenium (Se) toxicity impaired rapeseed growth and biomass production, decreased photosynthesis, and lowered photosynthetic pigment content. All these parameters were remarkably alleviated by MEL application. MEL significantly reduced cellular membrane damage and ROS formation. MEL effectively improved proline level, metabolite content, antioxidant enzymes, and their gene expression levels in *Brassica napus* under Se toxicity (Ulhassan et al., 2019). In another study, MEL treatment considerably enhanced net photosynthetic rate, growth traits, leaf gas exchange elements, and maintained macro- and micro-nutrient content in pepper leaves under B toxicity (Sarafi et al., 2017). MEL application improved antioxidant enzyme activities and root growth and reduced the MDA and proline levels in the roots of melon seedlings under Cu toxicity (Hu et al., 2020). In addition, MEL further enhanced the antioxidant enzyme system, along with a decline in the ROS level in spinach seedlings (Moussa and Algamal, 2017). MEL positively regulates the growth of tobacco under lead stress (Kobylińska et al., 2017), radish under Cd toxicity (Xu et al., 2020), and pepper under boron stress (Sarafi et al., 2017). **Table 5** provides an overview of the protective functions of MEL in horticultural crops under heavy metal toxicity.

**Table 5:** Melatonin mitigates heavy metals toxicity in horticultural plants.

Species name	Stress treatment	MEL level	Observation	References
Watermelon	40 mg vanadium	0.1 μM	Higher chlorophyll content, reduced MDA content, enhanced antioxidant enzymes, low vanadium uptake from root to shoot	Nawaz et al. (2018)
Cucumber	80 μM copper	10 nM	Promoted seedling growth, improved antioxidant enzymes. Inhibited ROS accumulation,	Cao et al. (2019)
Radish	200 mg lead	50 μM	Enhanced growth, reduced oxidative damage lowered lead accumulation	Tang et al. (2021)
Strawberry	100 μM cadmium	100 μM	Enhanced seedling growth, antioxidant enzymes, anthocyanin, and chlorophyll content; decreased oxidative stress biomarkers	Wu et al. (2021)
Tomato	50 μM nickel	100 μM	Improved photosynthesis, metabolites content, antioxidant enzymes; reduced oxidative damage and lowered Ni accumulation from root to shoot	Jahan et al. (2020)
Pepper	100 μM boron	1 μM	Enhanced mineral nutrient content, and carotenoids content, lowered metal uptake from root to shoot	Sarafi et al. (2017)
Fava bean	5 μM arsenic	50 μM	Enhanced pigment content, gas exchange parameters, reduced ROS production and MDA level	Siddiqui et al. (2020b)
Eggplant	10 mg cadmium	150 μmol	Improved water use efficiency, gas exchange characteristics	Tang et al. (2015)
Cucumber	90 mg iron	100 μmol	Improved leaf photosynthetic efficiency, growth characteristics, and antioxidant enzymes, reduced ROS, MDA and EL level	Ahammed et al. (2020)

### Acid rain, sodic alkaline toxic, and chemical stress

The protective role of MEL against acid rain, toxic chemicals, and sodic alkaline stresses has also been proven. Under acid rain conditions, the application of MEL on tomato plants leads to significant reductions in levels of MDA and H_2_O_2_, repairing the chloroplast’s grana lamella, along with escalations in growth parameters, antioxidant enzymes, pigment molecules, phenolic, flavonoids, and proline content (Debnath et al., 2018a). Debnath et al. (2018b) revealed that under stimulated acid-rain stress, the application of MEL to tomato plants exhibited significant improvements in yield attributes and quality traits of fruit. Moreover, pretreatment with MEL led to an increase in antioxidant enzymes’ activities, total soluble solids, soluble proteins, flavonoids, phenolics, and carotenoid content, along with remarkable reductions in MDA levels. MEL application remarkably improved the activity of antioxidant enzymes, chlorophyll content, and growth, and decreased the production of H_2_O_2_ and MDA in fenugreek under lead and acid rain stress (Xalxo and Keshavkant, 2019). Furthermore, Liu et al. (2015b) revealed that application of MEL under alkaline stress caused reductions in Na^+^ levels and enhanced the levels of K^+^ in tomato leaves, exhibiting the role of MEL in the maintenance of ion homeostasis and thus increasing the stress resistance of tomato plants to alkaline stress. The positive role of MEL in the stress resistance of plants to alkaline stress is due to the regulation of enzyme activity, and polyamine biosynthesis (Gong et al., 2017). MEL enhanced the photosynthetic pigment content of pea plants under paraquat stress (Szafrańska et al., 2017). In cucumber seedlings, the application of MEL under cinnamic acid stress increases plant stress resistance, mainly by integrating the morphology, mineral nutrient contents, and signaling crosstalk of plant hormones (Li et al., 2017c). The growth of cucumber plants is boosted by the application of MEL, which modulates mineral nutrient composition and nitrogen metabolism under nitrate stress (Zhang et al., 2017b).

## Future perspectives

The review will also help and encourage plant researchers to deeply examine the mechanism of stress tolerance mediated by MEL. Exogenously applied MEL is declared a potential growth regulator for plants, which aids under abiotic stress conditions by increasing plants’ growth, yield, and quality. Various functions of plants are found to be associated with MEL, such as the regulation of physiological functions, including seed germination and seedling growth, along with the functions of MEL in stress resistance under environmental stressors. The harmful effects of abiotic stresses are alleviated by the application of MEL, either directly by scavenging ROS or indirectly by improving the photosynthetic machinery, enhancing the activities of antioxidant enzymes, regulating metal transport and growth regulators in plants, as well as increasing osmotic metabolites. Although MEL has attracted the interest of plant researchers recently and progress can be seen on the topic, there are still unexplored MEL signaling pathways that, though complicated, need to be investigated under abiotic stresses. There exists a major gap in the literature about understanding the regulation of pathways by MEL and associated genes. Moreover, there is a need to address several major problems. For instance, there is a lack of understanding regarding the mechanisms of HM uptake, sequestration, and transportation as regulated by MEL. Future research should thus aim to deeply investigate the functions of MEL and its underlying mechanisms to sustain crop production under abiotic stress environments. 

## Author contributions

JZ: Conceptualization, literature survey, figure designing, writing major original draft, review structure. JH: Literature survey, writing—review and editing. All authors listed have made a substantial, direct, and intellectual contribution to the work and approved it for publication.
